# Inhibition of endoplasmic reticulum chaperone protein glucose-regulated protein 78 potentiates anti-angiogenic therapy in renal cell carcinoma through inactivation of the PERK/eIF2α pathway

**DOI:** 10.18632/oncotarget.5397

**Published:** 2015-10-12

**Authors:** Kyung Seok Han, Na Li, Pater A. Raven, Ladan Fazli, Sebastian Frees, Susan Ettinger, Ki Chung Park, Sung Joon Hong, Martin E. Gleave, Alan I. So

**Affiliations:** ^1^ Vancouver Prostate Centre and Department of Urologic Science, University of British Columbia, Vancouver, British Columbia, Canada; ^2^ Department of Urology and Urological Science Institute, Yonsei University College of Medicine, Seoul, Korea

**Keywords:** renal cell carcinoma, sunitinib, hypoxia, glucose-regulated protein 78, endoplasmic reticulum response

## Abstract

Tumor microenvironments are characterized by decreased oxygen and nutrition due to the rapid and progressive nature of tumors and also stresses induced by several anti-tumor therapies. These intense cell stressors trigger a protective cell survival mechanism heralded by the unfolded protein response (UPR). The UPR is induced by an accumulation of unfolded proteins in the endoplasmic reticulum (ER) following cell starvation. Although the ER stress response is implicated in cytoprotection, its precise role during anti-angiogenic therapy remains unclear. One of the major proteins involved in ER stress is glucose-regulated protein 78 (GRP78), which binds to unfolded proteins and dissociates from membrane-bound ER stress sensors. To determine the role of ER stress responses during anti-angiogenic therapy and the potential role of GRP78 in combined therapy in renal cell carcinoma (RCC), we used GRP78 overexpressing or knockdown RCC cells under hypoxic or hypoglycemic conditions *in vitro* and in animal models treated with sunitinib. Here, we report that GRP78 plays a crucial role in protecting RCC cells from hypoxic and hypoglycemic stress induced by anti-angiogenic therapy. Knockdown of GRP78 using siRNA inhibited cancer cell survival and induced apoptosis in RCC cells *in vitro* and also resulted in ER stress-induced apoptosis and hypoxic/hypoglycemic stress-induced apoptosis by inactivating the PERK/eIF-2α pathway. Finally, GRP78 knockdown showed potent suppression of tumor growth and enhanced the antitumor effect of sunitinib in RCC xenografts. Our findings suggest that GRP78 may serve as a novel therapeutic target in combination with anti-angiogenic therapy for the management of RCC.

## INTRODUCTION

Recently developed targeted therapies have shown promising results in the treatment of advanced malignancies. In particular, anti-angiogenic therapies that target vascular endothelial growth factor (VEGF) signaling have demonstrated remarkable clinical benefits in patients with advanced renal cell carcinoma (RCC) [1–3]. Targeting tumor angiogenesis induces severe tumor starvation, at which point, oxygen and nutrition deprivation successfully inhibits tumor growth and leads to disease stabilization. Sunitinib, one of the most efficacious multi-tyrosine kinase inhibitors targeting VEGF, significantly prolongs progression-free survival in patients with advanced RCC and is a first-line therapy for metastatic RCC [1]. However, anti-angiogenic therapy is generally not cytotoxic but rather cytostatic; thus, tumors shrink and regress, but many tumor cells survive anti-angiogenic treatment and resume growth following an interval of stability. Once the tumor acquires resistance to the anti-angiogenic agent, the tumor rapidly progresses and the prognosis is very poor, even with second- and third-line treatment options.

Tumors are often exposed to harsh microenvironments. Due to their rapid and progressive nature, tumors are surrounded by limited resources, such as oxygen and nutrients. Moreover, many cancer therapeutics deprive tumors of essential resources for cancer cell survival. These intrinsic and extrinsic environmental stressors generally induce the accumulation of unfolded proteins in the endoplasmic reticulum (ER) of cancer cells [4–5]. There, they elicit the unfolded protein response (UPR), a general cellular defense mechanism that dampens non-essential global protein synthesis to protect cells from stress. Many ER-resident proteins display altered expression patterns in cancer.

One of the most abundant ER-resident proteins is the ER chaperone glucose-regulated protein 78 (GRP78), also known as immunoglobulin heavy-chain-binding protein (BiP) [6]. A major UPR response is the induction of GRP78/BiP, which is highly expressed in a variety of tumors, including prostate cancer, melanoma, and head and neck cancer [7], and confers drug resistance in both proliferating and dormant cancer cells. GRP78 binds to unfolded proteins and dissociates from the membrane-bound ER stress sensors; dissociation of GRP78 allows the subsequent activation of stress sensors, which suppresses global mRNA translation to protect cells from excessive amounts of unfolded proteins [8]. This adaptive response activated by the accumulation of unfolded proteins in the ER is mediated by at least three ER proximal sensors: PKR-related ER kinase (PERK), inositol requiring enzyme-1 (IRE1) and activating transcription factor (ATF) 6 [9]. PERK is a serine/threonine kinase that phosphorylates eukaryotic translation initiation factor 2A (eIF2a), a translation initiation factor that functions in the early steps of protein synthesis by forming a ternary complex with GTP and initiator tRNA during stress and consequently attenuates global protein translation [9]. A recent study revealed that GRP78 is highly expressed in RCC cells and suggested a significant relationship between GRP78 expression and clinicopathological features of RCC, including tumor size, histological grade, and stage [10]. Another study showed that GRP78 expression was significantly associated with shorter disease-specific survival [11]. In addition, anti-vascular and anti-angiogenic therapies reportedly resulted in severe glucose and oxygen deprivation, which could lead to GRP78 induction in residual tumor cells and ultimately drug resistance [12].

GRP78 protects tumor cells from apoptosis under several stress conditions, including hypoxia, nutrient deprivation and cytotoxic chemotherapy [13–15]. Moreover, GRP78 expression is also a predictor of poor outcomes in RCC. However, the precise role of GRP78 in cancer following anti-angiogenic therapy remains unclear. We hypothesized that stress conditions induced by intrinsic or extrinsic stresses, such as anti-angiogenic therapy, lead to ER stress responses with increased GRP78 activity that aids tumor cell survival during oxygen and nutrient deprivation. In this study, we show that ER stress responses are activated during anti-angiogenic therapy and that inhibition of the UPR by GRP78 knockdown potently suppresses tumor progression and enhances the antitumor effects of anti-angiogenic therapy in RCC both *in vitro* and *in vivo*. Our results suggest that GRP78 may serve as a novel therapeutic target in the current therapeutic strategy for RCC.

## RESULTS

### GRP78 expression is highly upregulated by hypoxic or hypoglycemic stress in RCC cells

**Figure 1:**
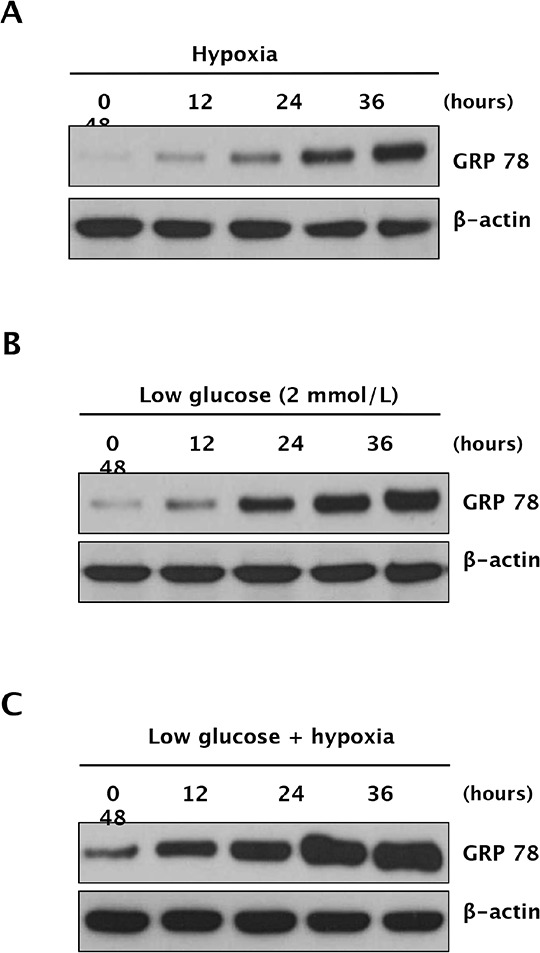
Expression of GRP78 under hypoxic and glucose-deprivation conditions in RCC cells Caki-1 cells were exposed to hypoxia (1% O_2_), low glucose, or both (hypoxia plus low glucose) for 48 h. Cell lysates were harvested at 0, 12, 24, 36, and 48 h, at which point, time-dependent GRP78 protein expression was analyzed under hypoxic conditions **A.** hypoglycemic conditions **B.** or both **C.** by Western blot analysis.

To further determine whether anti-angiogenic therapy-induced ER stress is caused by hypoxic stress, hypoglycemic stress, or a combination of both, we examined GRP78 expression in Caki-1 cells following exposure to hypoxic and/or hypoglycemic conditions. Western blot analysis indicated that GRP78 was upregulated by hypoxia, hypoglycemia, and both in a time-dependent manner (Figure 1A–1C). These data suggest that hypoxia and nutrition deprivation induce ER stress in RCC cells.

### Anti-angiogenic therapy induces ER stress response in RCC xenografts

**Figure 2:**
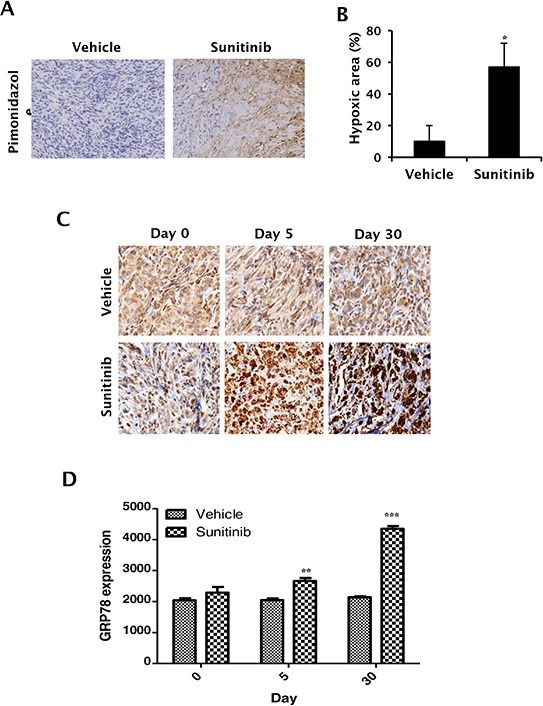
*In vivo* expression of GRP78 following sunitinib treatment in RCC xenografts **A–B.** Caki-1 tumor xenografts were treated with sunitinib (40 mg/kg) or vehicle. Hypoxic areas were assessed by pimonidazole immunohistochemical staining after 30 days of treatment. (A) Representative photographs were obtained using a light microscope (20 × magnification). (B) Hypoxic areas were quantitatively measured using ImageJ software. *P < 0.001 vs. vehicle. C–D, Caki-1 xenografts were treated with sunitinib for 30 days. GRP78 expression was then analyzed in re-treatment, 5-day treatment, and 30-day treatment tumor tissues. **C.** Representative photographs were taken using a light microscope (20 × magnification). **D.** Expression of immunostained GRP78 protein was quantitatively measured using MetaMorph 4.6 software (Universal Imaging Co., Downingtown, PA, USA). **P < 0.01 vs. vehicle, ***P < 0.01 vs. vehicle.

To determine whether ER stress chaperone protein GRP78 is upregulated during anti-angiogenic therapy, we treated Caki-1 xenografts with sunitinib, a multi-tyrosine kinase inhibitor and potent anti-angiogenic agent, and used immunohistochemical staining to examine changes in GRP78 expression in xenografts. Sunitinib treatment induced intratumoral hypoxia in Caki-1 xenografts (Figure 2A and 2B). After five days of sunitinib treatment, GRP78 expression was higher than that in the control group (Figure 2C and 2D). GRP78 was also upregulated in the control group at a later stage; however, the treatment group still displayed higher GRP78 expression (Figure 2C and 2D), suggesting that ER stress response may be induced by oxygen or nutrient deprivation from anti-angiogenic therapy. However, GRP78 expression was not significantly altered in normal tissues following treatment with sunitinib (Figure S1). Comparison of tumor and normal tissues following anti-angiogenic therapy revealed that GRP78 was more highly upregulated in tumor tissues. These data suggest that GRP78 may serve as a therapeutic target of anti-angiogenic therapy in RCC.

### Induction of GRP78 protects RCC cells from apoptosis through PERK/eIF2α signaling

**Figure 3:**
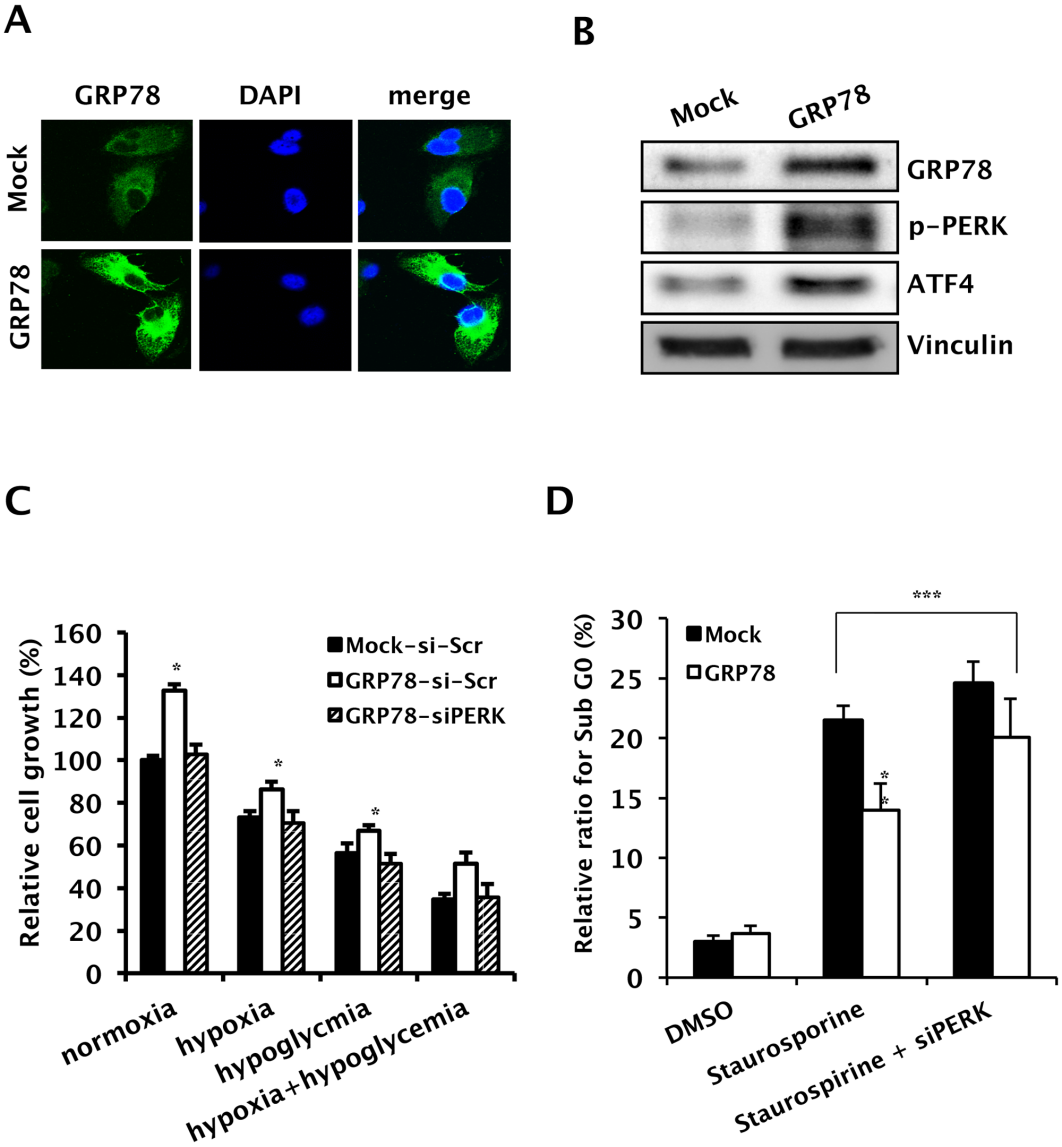
Pro-survival and anti-apoptotic roles of GRP78 overexpression though PERK/eIF2α signaling in RCC cells Caki-1 cells were stably transfected with pHR-CMV-GRP78 or mock vectors. **A.** Representative photographs showing overexpression of GRP78 in Caki-1-GRP78 relative to Caki-1-Mock cells. **B.** Changes in the expression of GRP78 downstream effectors. Whole-cell lysates from Caki-1 cells transfected with pHR-CMV-GRP78 or control vectors were subjected to Western blotting to examine the expression of phosphorylated PERK and ATF-4. Vinculin was used as a loading control. **C.** Cell growth was assessed before and after knockdown of PERK in GRP78-overexpressing Caki-1 cells compared to parental cells. Cell growth was measured using a crystal violet assay. *P < 0.01 vs. Mock-siScr. **D.** Cell cycle distribution was analyzed in GRP78-overexpressing Caki-1 cells before and after knockdown of PERK using FACS with PI staining. **P < 0.01 vs. Mock, ***P > 0.05.

To confirm the role of GRP78 in tumor cell survival and proliferation under stress conditions, we transfected Caki-1 cells with GRP78-encoded lentivirus (Caki-1-GRP78) or empty vector lentivirus (Caki-1-Mock). Immunofluorescence imaging showed that GRP78 was stably expressed at a higher level in Caki-1-GRP78 cells than in Caki-1-Mock cells (Figure 3A). Western blot analysis of proteins downstream of GRP78 revealed that GRP78 upregulation activated PERK through phosphorylation and increased ATF-4 (Figure 3B). We next performed a cell growth assay under hypoxic and/or hypoglycemic conditions, representing intratumoral stress conditions induced by anti-angiogenic therapy. Cell proliferation was enhanced in GRP78-overexpressing cells during hypoxia or hypoglycemia but these effects were removed by knockdown of PERK using PERK siRNA (Figure 3C). To further determine whether GRP78 protects tumor cells from apoptotic stress, apoptosis was induced by treatment with staurosporine, and a reduction in apoptotic cell death was confirmed in GRP78-overexpressing Caki-1 cells. Next, we knocked down PERK in GRP78-overexpressing Caki-1 cells using PERK siRNA plus staurosporine treatment. GRP78 overexpression did not affect apoptotic cell death after knockdown of PERK in Caki-1 cells (Figure 3D), indicating that GRP78 exerts both pro-survival and anti-apoptotic roles under conditions of stress by activating the PERK pathway in RCC cells.

### GRP78 knockdown suppresses tumor proliferation by inducing apoptosis in RCC cells

**Figure 4:**
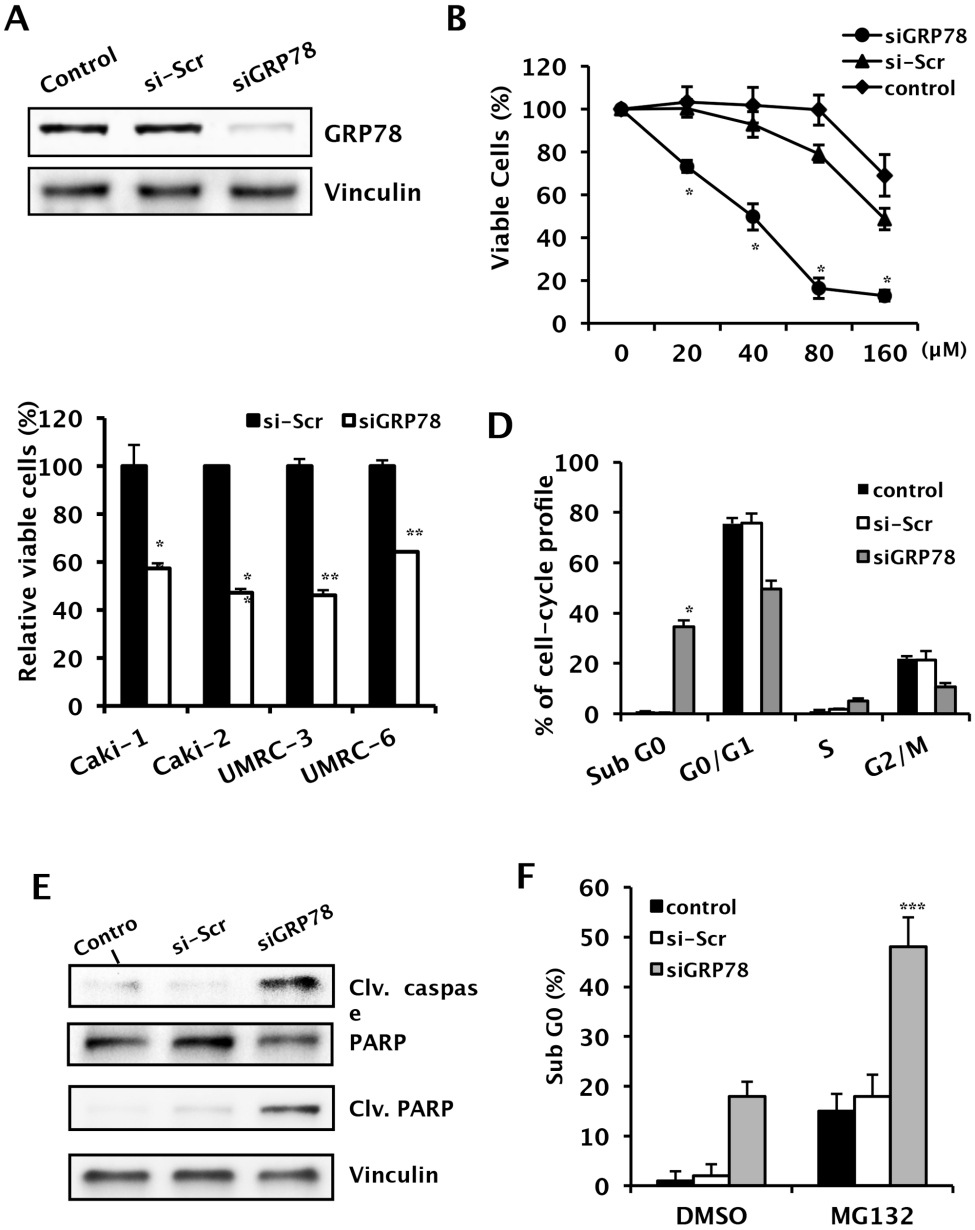
Effects of GRP78 knockdown on tumor growth and downstream effectors of ER stress pathways in RCC cells **A.** After RNA interference using siGRP78, whole cell lysates were subjected to Western blot analysis to evaluate the effectiveness of GRP78 knockdown in Caki-1 cells. Vinculin was used as a loading control. **B.** A cell growth assay was performed following GRP78 knockdown using various concentrations of siRNA to rule out the off-target cytotoxic effects of siRNA in Caki-1 cells, *P < 0.001. **C.** Growth of a wide panel of RCC cells following transient knockdown of GRP78. Four RCC cell lines were used for screening purposes. Cell growth was measured using a crystal violet assay following transient transfection with siGRP78, control siRNA, or reagent control. **P < 0.001 vs. scramble siRNA. **D.** Cell cycle analysis of Caki-1 cells treated with siGRP78. Cell cycle distribution was analyzed in Caki-1 cells treated with siGRP78 or control siRNA using FACS with PI staining. *P < 0.001 vs. si-Scr. **E.** Changes in cleaved caspase (Clv. caspase) and cleaved PARP (Clv. PARP) were analyzed in Caki-1 cells by Western blot analysis to confirm GRP78 knockdown-induced apoptosis. **F.** Cell cycle distribution was analyzed in Caki-1 cells using FACS with PI staining, combined with MG132 treatment and siGRP78 in Caki-1 cells, ***P < 0.001 vs. DMSO (siGRP78).

To study the inhibitory effect of GRP78 on RCC cell proliferation, we used GRP78 siRNA to transiently knock down GRP78 expression by >70% in all RCC cell lines (Figure 4A). GRP78 knockdown inhibited tumor proliferation in all RCC cell lines (Figure 4B and 4C). To evaluate the effect of GRP78 knockdown on the cell cycle, we examined cell cycle distribution by flow cytometry of propidium iodide-stained Caki-1 and UMRC-3 cells. GRP78 knockdown significantly induced apoptosis in Caki-1 cells (Figure 4D and S2). Western blot analysis showed that both caspase-3 and PARP were activated by GRP78 knockdown (Figure 4E and S3). To determine whether GRP78 knockdown enhances ER stress-induced apoptosis, we used MG132, a proteosome inhibitor that induces apoptosis via the ER stress-mediated apoptotic pathway [16], to induce ER stress in Caki-1 cells. MG132 inhibited cell growth and induced apoptosis in Caki-1 cells and GRP78 knockdown enhanced MG132-induced apoptosis (Figure 4F). These data demonstrate that GRP78 knockdown suppresses cancer cell survival by inducing apoptosis in RCC cells.

### GRP78 knockdown provokes apoptotic cell death under conditions of hypoxic and hypoglycemic stress by downregulating the PERK/ eIF2α/AFT-4 pathway

**Figure 5:**
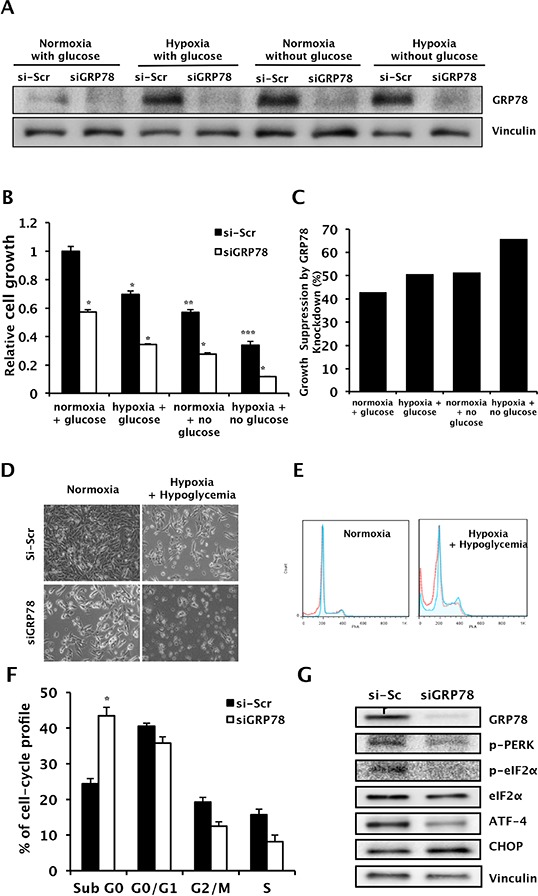
Changes in cell survival and apoptosis during hypoxia and hypoglycemia by GRP78 knockdown in RCC cells **A.** Whole cell lysates following transient knockdown of GRP78 using siGRP78 were subjected to Western blot analysis to confirm suppression of upregulated GRP78 during hypoxic and hypoglycemic conditions. Vinculin was used as a loading control. **B** and **C.** Cell growth was evaluated in Caki-1 cells using a crystal violet assay following GRP78 knockdown during hypoxic and/or hypoglycemic conditions. *P < 0.001 vs. si-Scr, **P < 0.001 vs. siScr in normoxia+glucose, ***P < 0.0001 vs. siScr in normoxia+glucose. (C) Relative growth suppression was calculated by internal comparison. **D.** Representative photos of cell growth were taken, 20X magnification. **E** and **F.** Cell cycle distribution was analyzed in Caki-1 cells treated with siGRP78 or control siRNA during hypoxic and hypoglycemic conditions using FACS with PI staining. *P < 0.001 vs. siScr. **G.** Western blot of proteins extracted from Caki-1 cells treated with either siGRP78 or scrambled siRNA following exposure to hypoxic and hypoglycemic stress. Vinculin was used as a loading control.

We next evaluated the effect of GRP78 knockdown on the survival of RCC cells under hypoxic and hypoglycemic conditions. Cell growth was inhibited by hypoxia and hypoglycemia. GRP78 siRNA successfully knocked down GRP78 expression induced by hypoxia and hypoglycemia (Figure 5A). GRP78 knockdown induced further growth inhibition in addition to that due to hypoxia, hypoglycemia, or both (Figure 5B–5D). To further study the effect of GRP78 inhibition combined with hypoxia or hypoglycemia on cell survival, we examined cell cycle distribution using flow cytometry. Cell cycle analysis showed that GRP78 knockdown significantly increased the number of apoptotic cells under all conditions examined (Figure 5E and 5F). GRP78 knockdown in cells suffering from hypoxic and hypoglycemic stress induced downregulation of phosphorylated PERK and eIF2α, inactivation of ATF-4, and activation of CHOP, suggesting that GRP78 further induces apoptotic cell death in RCC cells via downregulation of the PERK/eIF2α/AFT-4 pathway under conditions of stress (Figure 5G)

### *In vivo* knockdown of GRP78 induces tumor regression and enhances the anti-tumor effect of anti-angiogenic therapy in RCC xenografts

**Figure 6:**
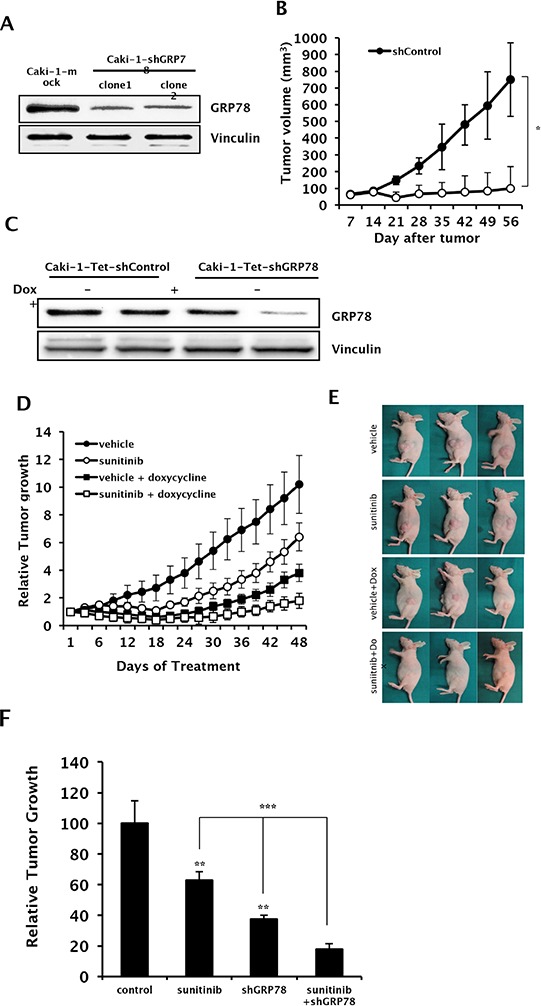
*In vivo* effects of GRP78 knockdown with sunitinib treatment on tumor growth in RCC xenografts **A.** Cell lysates were harvested from Caki-1 cells stably expressing shGRP78 or control vector and subjected to Western blot analysis to assess knockdown efficiency. Vinculin was used as a loading control. **B.** Tumor growth of xenografts from Caki-1 cells stably expressing shGRP78 or control vector were evaluated by measuring tumor size each week (*n* = 12 mice per group). *P < 0.001 vs. shControl. **C.** Cell lysates were harvested from Caki-1 cells stably expressing tetracycline-inducible shGRP78 or control vector and subjected to Western blot analysis to assess knockdown efficiency induced by doxycycline. Vinculin was used as a loading control. **D–F.** Xenografts were developed in nude mice using Caki-1 cells stably expressing tetracycline-inducible shGRP78 or control vector. Tumor size was measured every three days following treatment with doxycycline or vehicle combined with sunitinib or vehicle (*n* = 6 mice per group). (E) Representative photos of tumor xenografts were taken before sacrifice. (F) Relative tumor suppression in each treatment group compared to control. **P < 0.001 vs. vehicle, ***P < 0.001.

To confirm the effect of GRP78 inhibition on tumor growth *in vivo*, we developed Caki-1 cells stably expressing siGRP78 (Caki-1-shGRP78) or empty vector (Caki-1-Mock), which were then used to establish subcutaneous xenografts in mice (Figure 6A). Tumors were successfully developed, and consistent tumor growth was achieved in Caki-1-Mock xenografts. However, tumor growth was not successfully achieved in Caki-1-shGRP78 xenografts (Figure 6B, data for clone 2 not shown). These results suggest that GRP78 plays a vital role in tumorigenesis and tumor progression in RCC. Next, to determine whether GRP78 targeting has a therapeutic effect in RCC and whether GRP78 inhibition makes RCC cells more vulnerable to anti-VEGF-targeted therapy, we developed tetracycline-inducible shGRP78-expressing Caki-1 cells and established subcutaneous xenografts using these cells in nude mice (Figure 6C). Mice treated with doxycycline showed significantly reduced tumor growth compared with the other groups. Remarkable growth inhibition was observed using the combination of sunitinib and GRP78 suppression (Figure 6D–6F). These results indicate that GRP78 inhibition enhances antitumor activity of anti-angiogenic therapy *in vivo*.

## DISCUSSION

VEGF-targeted anti-angiogenic therapy is a revolutionary treatment for the treatment of advanced RCC. In contrast to conventional cytotoxic therapy, anti-angiogenic therapy suppresses tumor cells by inducing tumor hypoxia and nutrient deprivation. Nevertheless, cancer cells eventually develop compensatory mechanisms to survive starvation and hypoxia. One response to anti-angiogenic therapy is the ER stress response, which allows tumor cells to survive hypoxia, low-nutrient conditions, or low-pH conditions during anti-angiogenic therapy. GRP78 plays a central role in this defense mechanism and is conservatively preserved in normal cells. However, a previous study showed that GRP78 heterozygosity impedes mammary tumor growth and inhibits tumor proliferation. GRP78 heterozygosity also promotes cancer cell apoptosis while mice exhibit normal growth, organ development, and antibody production, suggesting that GRP78 may represent a good target candidate for cancer therapy [7]. Here, we showed that GRP78 expression was induced in RCC xenografts following anti-angiogenic therapy, and that GRP78 knockdown effectively inhibited tumor proliferation and induced apoptosis in RCC both *in vitro* and *in vivo* by impeding UPR pathways.

The anti-tumor effects of anti-angiogenic agents are attributable to their ability to deprive tumors cells of vessels supplying oxygen and nutrients. Tumor cells can adapt to a low-oxygen environment, owing to hypoxia-inducible factors (HIFs), which regulate the expression of more than 60 genes involved in angiogenesis, anaerobic glycolysis, and cell survival; the coordinated expression of these genes results in cellular adaptation to prolonged and acute hypoxia [17–19]. However, when the downstream effectors of HIFs are functionally blocked, hypoxia induces additional HIF-independent adaptive responses, such as ER stress, that contribute to increased survival under low-oxygen conditions. Thus, cells with a compromised ER stress pathway show significant sensitivity to ER stress created by the deleterious effects of accumulated unfolded proteins in the ER [17, 20, 21].

Since GRP78 is a master regulator of ER stress, UPR responses can be effectively downregulated by targeting GRP78. Hypoxic stress induces ER stress responses in a PERK- and eIF2α-dependent manner. UPR is orchestrated through activation of PERK, IRE1 and ATF6, all three of which are bound and sequestered by GRP78 under normal conditions [22]. When an unfolded protein load triggers the dissociation of GRP78 from these three sensors, UPR signaling is initiated. Activated PERK rapidly increases phosphorylation of the translation initiation factor eIF2α, which prevents the influx of additional nascent polypeptides to the ER [22] and upregulates genes that promote amino acid sufficiency and redox homeostasis [23–25]. Phosphorylation of eIF2α plays a central role in coupling the rate of protein synthesis to the cellular response to different types of stress and energy availability. A rapid and robust increase in eIF2α phosphorylation at Ser51 is observed in response to hypoxia [26]. This modification correlates with a reduction in protein synthesis rate and is not observed following other stresses, such as low serum or genotoxic stress. Phosphorylation of eIF2α is independent of HIF1α status, as it also occurs in a similar manner in HIF1α^−/−^ mouse embryonic fibroblasts [9, 27]. In the present study, inhibition of GRP78 reduced the PERK phosphorylation in RCC cells. In addition, GRP78 knockdown inhibited the activation of both PERK and eIF2α under both normoxic and hypoxic conditions, suggesting that GRP78 may be the best target for inhibiting the ER stress response.

CHOP is downstream of the PERK and eIF-2α phosphorylation cascade. Activation of PERK and eIF2a induces CHOP accumulation, which plays a critical role in the switch from pro-adaptive to pro-apoptotic signaling [9]. CHOP promotes protein synthesis and oxidation in the stressed ER, thereby contributing to the induction of cell death. It also modulates the Bcl-2 family of proteins and growth and arrest DNA damage inducible protein 34 (GADD34), causing damage to the mitochondrial membrane and the release of cytochrome *c* into the cytosol [28–30]. Our results show that the pro-apoptotic effects of GRP78 inhibition occur via CHOP activation, subsequent to PERK and eIF2α activation in RCC cells.

Inhibition of GRP78 halts the important defense mechanism required for survival in starving tumor cells and makes tumor cells more vulnerable to environmental stresses such as hypoxia and glucose deprivation. Combination therapy that stimulates ER stress-mediated apoptotic pathways while simultaneously downregulating the induction of pro-survival GRP78 may substantially enhance drug efficacy. For this reason, targeting ER stress responses through GRP78 inhibition may be a reasonable approach for combination cancer therapy with VEGF-targeted anti-angiogenic agents. In addition to the protective role of GRP78 in cancer cells, GRP78 also promotes tumor proliferation. In the present study, overexpression of GRP78 increased the proliferation of RCC cells, and GRP78 knockdown had significant antitumor effects under normal conditions without stresses (i.e., hypoxia and glucose deprivation). These results demonstrate that GRP78 plays a critical role in tumor proliferation and progression even in normal tumor environments, suggesting that GRP78 targeting may represent an effective cancer treatment option as monotherapy.

In conclusion, our study showed that the inhibition of GRP78 successfully enhanced the antitumor effect of sunitinib in RCC, indicating that GRP78 is an excellent target for combination therapy with anti-angiogenic treatments. Targeting ER stress responses may serve as a novel approach to enhance the therapeutic efficacy of anti-angiogenic agents and to delay the development of resistance.

## MATERIALS AND METHODS

### Cell culture and reagents

Two human RCC cell lines, Caki-1 and Caki-2, were obtained from the American Type Culture Collection (ATCC) and maintained in McCoy's 5A Medium (HyClone, Thermo Fisher Scientific) supplemented with 10% fetal bovine serum (FBS). Two additional human RCC cell lines, UMRC-3 and UMRC-6, were kindly provided by Dr. P. Black (Vancouver Prostate Centre, University of British Columbia, Vancouver, Canada) and maintained in Minimal Essential Medium (MEM; Invitrogen) supplemented with 10% FBS and 2 mmol/L L-glutamine. All cells were cultured at 37°C in a humidified atmosphere with 5% CO_2_. For all studies, cell lines were passaged for a maximum of two months.

### Transient transfections

To develop cells with transient GRP78 knockdown, Caki-1 and UMRC-3 cells were transiently transfected with a 19-bp small interfering RNA (siRNA) targeting GRP78 (sense: 5′-GGAGCGCAUUGAUACUAGA-3′, anti-sense: 5′-GCCUAGGUCUCUUAGAUGA-3′) or a non-silencing siRNA (si-Scr) [31]. Caki-1 and UMRC-3 cells were seeded onto 6-well plates at a density of 1.2 × 10^5^ cells/well. At 30%–50% confluence, cells were transfected for 16 h with the siRNAs using Lipofectamine 2000 (Invitrogen Life Technologies) diluted with OPTIMEM (Invitrogen Life Technologies) according to the manufacturer's instructions. After transfection, the medium was replaced with fresh medium, and cells were incubated for 48–72 h according to the purpose of the experiment. To develop cells with transient protein kinase-like ER kinase (PERK) knockdown, GRP78-overexpressing Caki-1 and parental Caki-1 cells were transfected with PERK siRNA (Santa Cruz Biotechnology).

### Cloning and stable transfections

The pHR-CMV lentivirus system was used as described previously to generate GRP78-overexpressing Caki-1 cells [31]. Briefly, human GRP78 cDNA was subcloned into the pHR-CMV lentiviral vector. HEK293T cells were then transiently transfected with GRP78-pHR-CMV, along with packaging vectors, VSVG and R8.91. Forty-eight hours after transfection, lentivirus-containing culture medium was collected and added to Caki cells. The pLKO.1 lentivirus system was used to develop Caki-1 GRP78 knockdown cells stably expressing shGRP78. Oligonucleotides for GRP78 small hairpin RNA (shRNA) were synthesized and cloned into pLKO.1 by Sigma Chemical Company. shGRP78-overexpressing Caki-1 clones were then selected with puromycin (Invitrogen). The pSingle-tTS-shRNA vector system (Clontech) was used to generate stable tetracycline-inducible knockdown of GRP78 in Caki-1 cells. GRP78-specific (sense: 5′-TCGAGGGGAGCGCATTGATACTAGATTCAAG AGATCTAGTATCAATGCGCTCCTTTTTTACGCG TA-3′, antisense: 5′-AGCTTACGCGTAAAAAAGGAGCGCATTGAT ACTAGATCTCTTGAATCTAGTATCAATGCGC TCCCC-3′) and control non-targeting shRNA sequences (sense: 5′-TCGAGG AGTTCAACGAGTATCAGCATTCAAGAGATG CTGATACTCGTTGAACTTTTTTTACGCGTA-3′, antisense: 5′-AGCTTACGCGTAAAAAAAGTTCAACGAG TATCAGCATCTCTTGAATGCTGATACTCGTT GAACTCC-3′) were designed following the manufacturer's protocol. Annealed, double-stranded shRNA oligonucleotides were cloned into the *XhoI*/*Hind*III site of the pSingle-tTS-shRNA vector. Cloning was confirmed by nucleotide sequencing. The pSingle-tTS GRP78 shRNA or pSingle-tTS non-silencing control shRNA was transfected into Caki-1 cells using Xtreme Gene 9 (Roche) diluted with OPTIMEM according to the manufacturer's instructions. Transfected cells were selected using 400 μg/mL G418 (Roche) and maintained in medium containing 200 μg/mL G418. Stable cell clones were then confirmed for doxycycline-induced GRP78 gene silencing by both Western blot analysis and reverse transcription-polymerase chain reaction (RT-PCR). After polyclonal selection and confirmation, stable cell lines were frozen at early (less than 10) passages.

### Development of hypoxia and glucose-deprivation

Hypoxic conditions (1% oxygen) were induced using a hypoxia chamber (Coy Corporation). Cells were exposed to hypoxia (1% oxygen) and maintained in the hypoxia chamber during the assay. Deprivation of glucose was achieved by using glucose-free media supplemented with low glucose (2 mmol/L).

### Cell viability assay

Cells were seeded onto six-well plates at 1.2 × 10^5^ cells per well, incubated overnight, and transfected. After incubation for an additional 72 h, cells were fixed with 1% glutaraldehyde and stained with a 0.5% crystal violet solution. Cells were then washed with water, and any remaining crystal violet was resolved with Sorensen's solution. Absorbance was measured at 562 nm on a spectrophotometer. All experiments were performed in triplicate.

### Cell cycle analysis and apoptosis

Cells were harvested and fixed with 70% alcohol as a single-cell suspension. Fixed cells were collected by centrifugation and washed with phosphate-buffered saline (PBS). Cells were incubated with RNase A (1 mg/mL) for 30 min at 37°C and then incubated with propidium iodide (PI; 20 μg/mL; Sigma Chemical) for 30 min. Relative DNA content was determined using a FACSCanto II flow cytometer and FACSDiva software (BD Bioscience).

### Western blot analysis

Cellular proteins were harvested in RIPA lysis buffer containing vanadate, phosphatase inhibitor and phenylmethanesulfonyl fluoride. Proteins were resolved by electrophoresis in polyacrylamide gels and transferred to a nitrocellulose membrane using a Trans-Blot system (Bio-Rad) as described previously [14]. Membranes were blocked in 5% skim milk diluted with Tris-buffered saline and Tween 20 (TBST) for 1 h at room temperature and incubated with primary antibodies diluted in 3% skim milk in TBST for 1 h at room temperature or overnight at 4°C. Antibodies against the following proteins were used for Western blot analysis (all from Santa Cruz Biotechnology): GRP78 (C–20) (diluted 1:1000), PERK (1:1000), p-PERK (1:250–500), p-eIF2α (1:250–500), GADD153 (1:500), CREB-2 (1:500), and vinculin (1:5000). Anti-goat, anti-rabbit, and anti-mouse IgG secondary antibodies (1:5000) were used. Amersham enhanced chemiluminescence reagents (GE Healthcare) or Supersignal chemiluminescence reagents (Thermo Scientific) were used to detect immunoreactive proteins.

### Immunofluorescence

Cells were seeded onto glass coverslips in six-well plates and allowed to attach overnight. To evaluate baseline expression of GRP78 in wild-type RCC cells and GRP78-overexpressing cells, cells were washed with ice-cold PBS, fixed with ice-cold methanol mixed with acetone (3:1) for 10 min at room temperature, and treated with 0.25% Triton X-100 in PBS for 10 min. After blocking with 3% bovine serum albumin for 30 min, cells were then incubated with anti-GRP78 (C–20) antibody at room temperature for 1 h, washed three times with TBS, and incubated with Alexa Fluor 488-conjugated anti-goat antibody for 1 h in a dark room. Nuclei were stained with DAPI. Immunofluorescence was imaged using a Zeiss confocal laser scanning microscope.

### *In vivo* xenograft models

All animal experiments were conducted in compliance with accepted standards of the University of British Columbia Committee on Animal Care. General anesthesia of mice was induced and maintained by inhaled isoflurane. Six- to eight-week-old female nu/nu nude mice were subcutaneously injected in the flank area with a cell suspension containing 3 × 10^6^ tumor cells. Caki-1 cells were used to evaluate GRP78 expression following sunitinib treatment. Caki-1 cells stably expressing doxycycline-inducible shGRP78 were used to investigate the effect of GRP78 knockdown *in vivo*. When the tumor size reached 150–250 mm^3^, mice were randomized to receive vehicle, sunitinib, doxycycline, or sunitinib and doxycycline for 30 days (*n* = 8 mice per group). Every three days, tumor size was measured using calipers and body weight was determined. Tumor volume was calculated as length × width × height × 0.5. After treatment was complete, mice were euthanized in a CO_2_ chamber followed by cervical dislocation. Tumors were fixed in 10% formalin for immunohistochemical staining or frozen at − 80°C for protein and RNA analyses.

### Immunohistochemistry

Formalin-fixed, paraffin-embedded tumors were cut into 4-μm sections, which were deparaffinized, rehydrated with xylene and ethanol, and transferred into 0.02% Triton for permeabilization. Slides in citrate buffer (pH 6) were heated in a steamer for 3 min, washed in PBS for 5 min, incubated in 3% bovine serum albumin, transferred into 3% H_2_O_2_, and incubated with anti-GRP78 antibody overnight. For quantitative analysis of immunohistochemical staining, expression of pimonidazole was measured using ImageJ and expression of GRP78 was measured using MetaMorph 4.6 software (Universal Imaging Co., Downingtown, PA, USA). Automated counting of numbers of pixels in the four different intensity zones were received and after determining these numbers, we applied them to a simple algebraic formula as shown below to determine the score of the image. Score = (Number of pixels in a zone) × (Score of the zone) / (Total number of pixels in the image).

### Statistical analysis

All experiments were performed at least three times. Results are presented as the means ± standard error (SE). Statistical analysis was performed using SigmaPlot 12 (Systat Software Inc., Chicago, IL, USA). Student's *t*-test was applied for two-group comparison. *P* values less than 0.05 were considered to indicate statistical significance.

## SUPPLEMENTARY FIGURES



## Abbreviations

UPR: unfolded protein response
ER: endoplasmic reticulum
GRP78: glucose-regulated protein 78
RCC: renal cell carcinoma
PER: PKR-related ER kinase
IRE1: inositol requiring enzyme-1
ATF: activating transcription factor
VEGF: vascular endothelial growth factor
PARP: Poly ADP ribose polymerase
HIFs: hypoxia-inducible factors
CHOP: C/EBP-homologous protein
GADD34: growth and arrest DNA damage inducible protein 34
ATCC: American Type Culture Collection
FBS: fetal bovine serum
MEM: Minimal Essential Medium
siRNA: small interfering RNA
PBS: phosphate-buffered saline
PI: propidium iodide
TBST: Tris-buffered saline and Tween 20
