# Supplementary material for: Inhibition of endoplasmic reticulum chaperone protein glucose-regulated protein 78 potentiates anti-angiogenic therapy in renal cell carcinoma through inactivation of the PERK/eIF2α pathway
Source: Oncotarget. 2015 Oct 12;6(33):34818–30. doi: 10.18632/oncotarget.5397 (PMC4741492; doi:10.18632/oncotarget.5397)
Supplement: Supplementary file 1 [file oncotarget-06-34818-s001.pdf]

SUPPLEMENTARY FIGURES

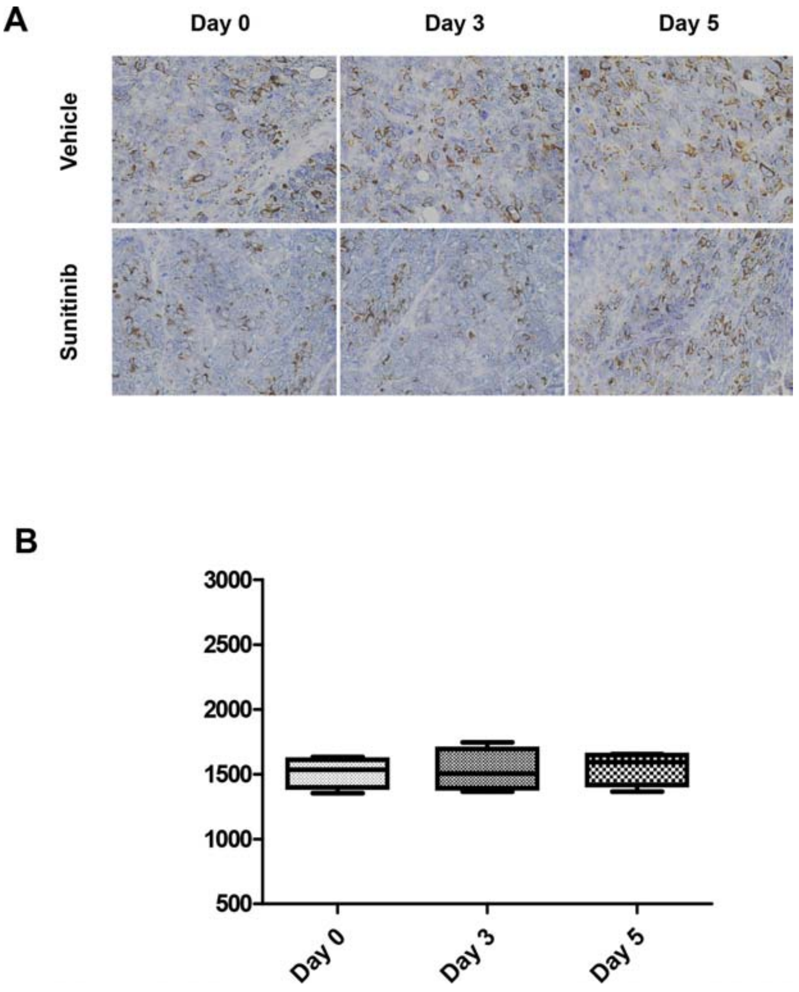

Supplementary Figure S1: Effect of sunitinib treatment on GRP78 expression in normal tissues. A. Immunohistochemical staining with GRP78 antibody on normal liver tissue before and after sunitinib treatment. Representative photographs were taken using a light microscope (20 × magnification). **B.** Expression of immunostained GRP78 protein was quantitatively measured using MetaMorph 4.6 software (Universal Imaging Co., Downingtown, PA, USA).

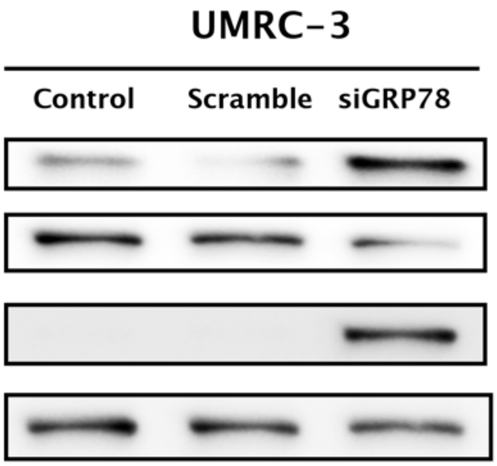

Supplementary Figure S2: Cell cycle distribution was analyzed in Caki-1 cells treated with siGRP78 or control siRNA using FACS with PI staining.

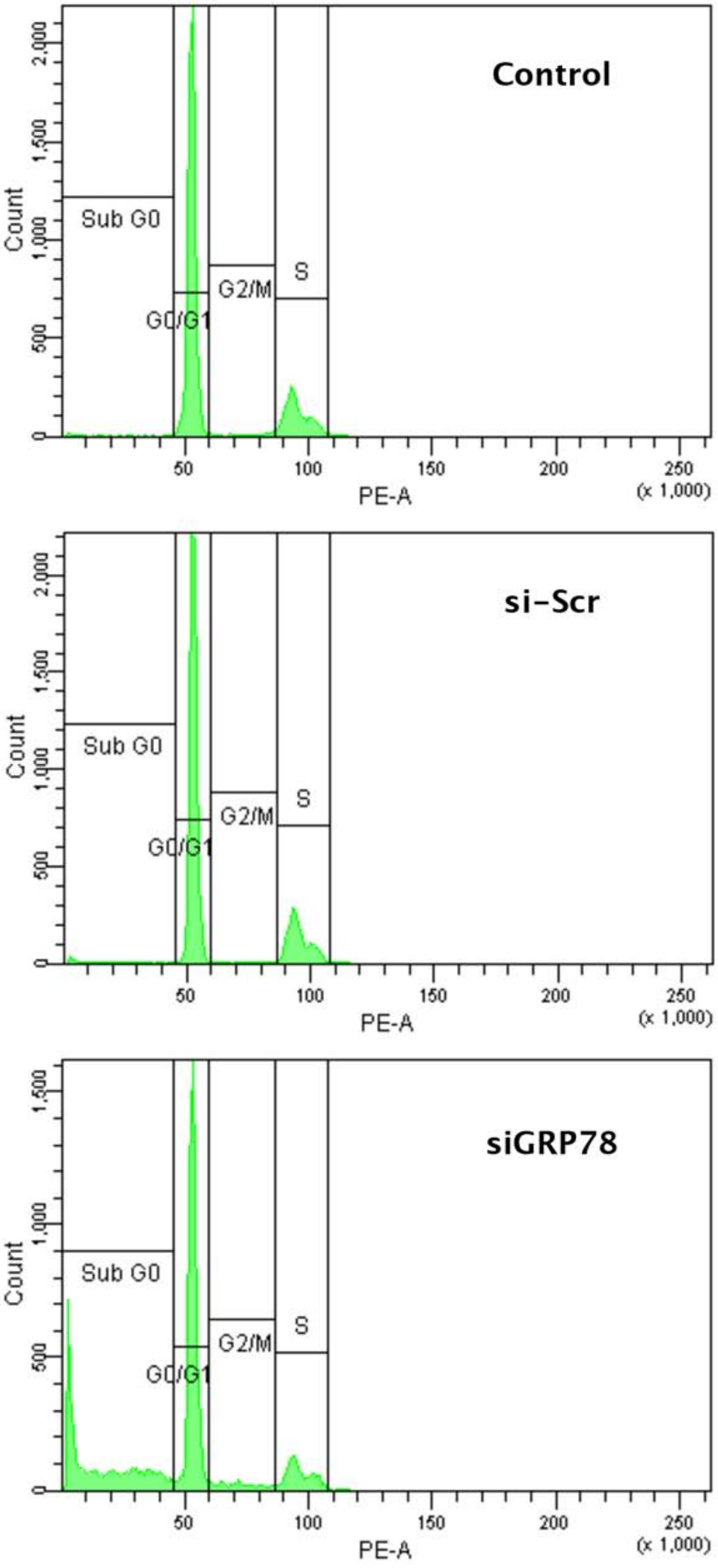

Supplementary Figure S3: Effects of GRP78 knockdown on apoptosis in UMRC-3 cells. Changes in cleaved caspase and cleaved PARP were analyzed in UMRC-3 cells by Western blot analysis to confirm apoptosis-induced knockdown of GRP78.
